# Association between HBV DNA levels and bone mineral density in antiviral-naive chronic hepatitis B patients

**DOI:** 10.3389/fendo.2026.1714943

**Published:** 2026-02-18

**Authors:** Xinyu Zhou, Xue Jing, Na Zu, Zhanghao Li, Xinjuan Kong, Shijin Wang

**Affiliations:** Department of Gastroenterology, The Affiliated Hospital of Qingdao University, Qingdao, Shandong, China

**Keywords:** bone health, bone mineral density, chronic hepatitis B, HBV DNA, osteoporosis

## Abstract

**Objectives:**

Recent studies have shown that patients with chronic hepatitis B (CHB) have an increased prevalence of osteoporosis. However, the direct relationship between hepatitis B virus (HBV) DNA (representing the viral replication level) and bone mineral density (BMD) remains undefined. We aimed to investigate the association between HBV DNA levels and BMD in middle-aged and elderly CHB patients without prior antiviral therapy.

**Methods:**

This cross-sectional study recruited 362 untreated patients with CHB (men aged ≥50 years and postmenopausal women) who underwent both HBV DNA testing and dual-energy X-ray absorptiometry (DXA) within a 6-week interval. Based on bone mineral status, patients were categorized into three groups: normal BMD, osteopenia, and osteoporosis. Multiple regression and generalized additive models (GAMs) were applied to analyze associations between HBV DNA and BMD, whereas Receiver operating characteristic (ROC) curve analysis was employed to evaluate the discriminatory ability of HBV DNA levels to distinguish patients with osteoporosis. Exploratory mediation analysis of β-CTX and CRP was performed to assess indirect statistical associations linking HBV DNA with BMD.

**Results:**

Patients with osteoporosis had considerably higher HBV DNA levels than those with osteopenia or normal BMD. In multivariable analyses, each 1 log_10_ IU/mL increase in HBV DNA was associated with a 0.22-unit decrease in BMD T-score in the total cohort, with a stronger inverse association observed in males (β = −0.32) than in postmenopausal females (β = −0.16). GAMs revealed a continuous negative association between HBV DNA levels and BMD across the spectrum from normal bone density to osteoporosis (P for trend <0.001). HBV DNA demonstrated good between-group discriminatory ability for differentiating osteoporosis, with superior accuracy in males (area under the ROC curve = 0.847; optimal cut-off: 2.857 log_10_ IU/mL). Exploratory mediation analyses suggested that β-CTX and CRP may contribute to the association between HBV DNA and BMD in the total cohort; however, only β-CTX demonstrated a notable mediating effect in males. Notably, the direct association between HBV DNA levels and BMD remained evident across all mediating models.

**Conclusion:**

Higher HBV DNA levels are independently associated with lower BMD and demonstrate discriminatory ability for osteoporosis status in patients with CHB. Longitudinal studies are warranted to determine the predictive value of HBV DNA in predicting osteoporosis risk.

## 1 Introduction

Chronic hepatitis B (CHB) remains a considerable global public health issue. The most recent epidemiological data indicate that approximately 254 million individuals worldwide are living with CHB infection, with an estimated 1.08 million deaths in 2022 attributable to hepatitis B virus (HBV) - associated complications (1). CHB infection is defined by the persistence of hepatitis B surface antigen (HBsAg) for more than six months, accompanied by positive immunoglobulin G antibody to hepatitis B core antigen (IgG anti-HBc). Serum HBV DNA, as a direct marker of viral replication, serves as an indicator of infectivity and as a critical parameter for monitoring therapeutic efficacy and assessing clinical outcomes (2, 3). Notably, untreated patients exhibit a vast dynamic range of HBV DNA levels (from <10 IU/mL to >10^9^IU/mL), and this heterogeneity can differentially influence the risk of extrahepatic complications (4).

Osteoporosis, characterized by reduced bone strength and increased fracture risk (5), is particularly prevalent among postmenopausal females and aging male populations (6). Notably, emerging evidence demonstrates a substantial association between osteoporosis and CHB infection (7, 8), particularly in patients with poor viral control (9). A cross-sectional study in the United States involving over 10,000 participants revealed negative correlations between HBV infection status and femoral/spinal bone mineral density (BMD) in male and postmenopausal female patients (10), while a Korean nationwide cohort study revealed a 19% increased osteoporosis risk in patients with CHB (11). A German retrospective analysis demonstrated sex-specific risks: female patients with CHB had a significantly higher risk of osteoporosis, whereas male patients had a higher risk of fractures (12). Consistent with these findings, Korean elderly patients with CHB with fractures had significantly higher serum HBV DNA levels compared to patients with CHB without fractures (13).

HBV replication may contribute to impaired bone metabolism through immune-inflammatory mechanisms. In particular, continuous HBV replication has been shown to provoke the release of pro-inflammatory cytokines, including tumor necrosis factor-α and interleukin-6 (14). These cytokines induce osteoclast differentiation and activation of the RANKL–NF-κB signaling pathway, thereby accelerating bone resorption and loss of BMD (15). C-reactive protein (CRP) is implicated as a functional contributor to bone resorption as well (16). In addition, chronic HBV-related hepatic inflammation may disturb the “hepato–bone axis”, impairing endocrine and metabolic pathways essential for skeletal homeostasis (17).

Although current evidence indicates a remarkable association between HBV infection and BMD reduction, the relationship between HBV DNA load and BMD remains poorly understood. Given that HBV DNA levels are routinely monitored in clinical practice, examining their potential association with BMD could enhance the identification of individuals presenting with osteoporosis. This cross-sectional study investigated the correlation between HBV DNA levels and BMD among middle-aged and elderly patients with untreated CHB; its aim was to examine the potential of HBV DNA as an indicator for identifying those patients at risk of osteoporosis and fracture, thereby contributing to the prevention of fracture-related disability.

## 2 Materials and methods

### 2.1 Study population

This single-center cross-sectional study initially enrolled 573 treatment-naive middle-aged and elderly patients with CHB (postmenopausal females and males ≥50 years) who underwent both HBV DNA testing and dual-energy X-ray absorptiometry (DXA) at Qingdao University Affiliated Hospital from January 2014 to January 2024. All participants were antiviral-naive at the time of the index assessment, defined as having never received nucleos(t)ide analog therapy before or at the time of HBV DNA testing and DXA. This study was designed as a cross-sectional analysis focusing on baseline virological status and BMD measured at the index assessment. Patients who met guideline-based criteria (18) at or after the index assessment were recommended to initiate antiviral therapy accordingly. The severity of liver disease was systematically assessed in all included patients based on imaging findings, clinical evaluation, and laboratory parameters. The majority of patients (349 cases) had CHB without cirrhosis. A small subset of patients (13 cases) had cirrhosis confirmed by ultrasound screening, and all of them were subsequently classified as compensated cirrhosis (Child–Pugh class A). None of these patients had a history of hepatic decompensation, including but not limited to ascites, variceal bleeding, hepatic encephalopathy, or jaundice.

**Figure 1:**
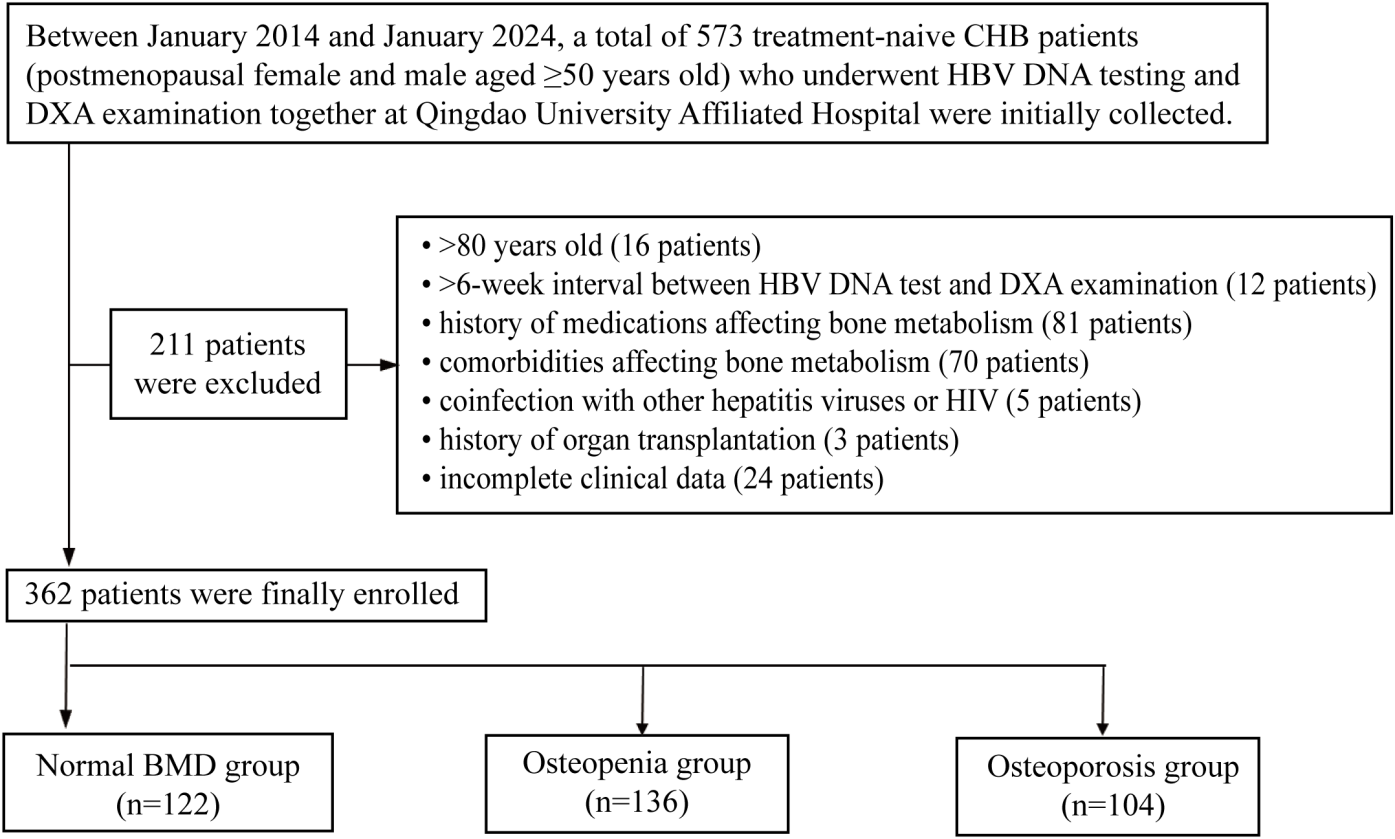
Study population screening flowchart.

After applying exclusion criteria: (1) age >80 years (n = 16); (2) >6-week interval between HBV DNA testing and DXA examination (19) (n = 12); (3) bone metabolism-affecting medications: antiosteoporotic agents (bisphosphonates, RANKL monoclonal antibodies, etc.) or osteoporosis-inducing drugs (glucocorticoids [≥2.5 mg daily for >3 months] (20), chemotherapy, etc.) (n = 81); (4) comorbidities affecting bone metabolism: osteoproliferative disorders (Paget’s disease, osteopetrosis, etc.) or osteodestructive conditions (hyperthyroidism, Cushing’s syndrome, Child–Pugh B/C cirrhosis, chronic kidney disease stage 5, etc.) (n = 70); (5) coinfection with other hepatotropic viruses or human immunodeficiency virus (HIV) (n = 5); (6) history of organ transplantation (n = 3); and (7) incomplete clinical data (n = 24), a total of 362 eligible patients were included in the final analysis and stratified by BMD measurements into three groups: normal BMD (n = 122), osteopenia (n = 136), and osteoporosis (n = 104) (**Figure 1**).

This retrospective study was approved by the Ethics Committee of Qingdao University Affiliated Hospital, and the requirement for informed consent was waived due to the use of deidentified data. The study adhered to the ethical principles of the Declaration of Helsinki.

### 2.2 Sample size

In the sample size calculation, serum HBV DNA level was set as the key variable, hypothesizing significant differences in HBV DNA levels among different BMD groups in middle-aged and elderly patients with CHB without prior antiviral treatment. Sample size estimation was performed using the G*Power 3.1 software with the following parameters: effect size (Cohen’s f² = 0.15), significance level (α = 0.05), and statistical power (1-β = 0.8). The final enrollment count was 362 patients, meeting the predetermined sample size requirement (a minimum of 303 cases).

### 2.3 Baseline data collection

The following clinical parameters were extracted from the electronic medical records of all enrolled patients with CHB: age, sex, body mass index (BMI), history of smoking/alcohol use, medical history of chronic diseases, and medication history; hepatitis serology, biochemical parameters, platelet count, CRP, and bone turnover markers (BTMs); BMD and T-scores at the lumbar spine (LS), femoral neck (FN), and total hip (TH), along with diagnoses of bone mass status. In addition, liver disease stage was assessed using imaging findings and clinical parameters, including the Child–Pugh score, the presence or absence of cirrhosis, and the presence or absence of decompensation. The Fibrosis-4 (FIB-4) index as a non-invasive score for liver fibrosis was calculated as age (years) × aspartate aminotransferase (U/L)/[platelet count (10^9^/L) × √alanine aminotransferase (U/L)] (21). Alcohol use was defined as current or previous consumption for at least six consecutive months with daily intake, and smoking was defined as current or previous cigarette use for at least six consecutive months on a daily basis.

### 2.4 Virological and bone markers

Virological serum markers, i.e., hepatitis B e antigen (HBeAg) and hepatitis B core antibody (HBcAb), were quantified using the Abbott Architect chemiluminescent microparticle immunoassay (CMIA) system, employing sandwich (HBeAg) and competitive (HBcAb) immunoassays. HBV DNA was measured using real-time PCR with a diagnostic kit (Shanghai Fosun Pharmaceutical Co., Ltd; lower limit of detection [LLOD]: 50 IU/mL; genotypes A-D). HBV DNA values (IU/mL) were log_10_-transformed for statistical analysis. For HBV DNA values below the LLOD, 25 IU/mL (LLOD/2) was imputed prior to log_10_ transformation to allow inclusion in continuous analyses and to minimize bias from left-censoring. Bone turnover markers—including β-C-terminal telopeptide of type I collagen (β-CTX), procollagen type I N-terminal propeptide (P1NP), N-terminal mid-fragment osteocalcin (N-MID), intact osteocalcin (OC), and parathyroid hormone (PTH)—were analyzed using Roche Cobas electrochemiluminescence immunoassay (ECLIA): β-CTX by competitive assay with ruthenium (Ru)-labeled analogs; others by sandwich immunoassays. Serum 25-hydroxyvitamin D [25(OH)D] was measured using the Abbott ARCHITECT CMIA. All methods maintained original analytical specifications.

### 2.5 BMD determination

BMD (g/cm²) at the LS (L1-L4), FN, and TH was measured using the PRIMUS DXA system (OSTEOSYS, Seoul, Korea). Before each measurement, a phantom test was performed to ensure accuracy. The DXA workstation software automatically calculated T-scores, expressed as standard deviations (SD) from the mean BMD of a race- and gender-matched young healthy reference population. According to World Health Organization (WHO) criteria, patients in this study were classified as follows: normal BMD with T-score ≥-1.0, osteopenia with T-score between -1.0 and -2.5, or osteoporosis with T-score ≤-2.5 (22). BMD status was determined using the lowest T-score among the LS (L1-L4), FN, and TH.

### 2.6 Statistical analysis

Normality of continuous variables was assessed using the Kolmogorov–Smirnov test. Normally distributed variables were expressed as mean ± SD and compared using one-way analysis of variance, while non-normally distributed variables were presented as median and interquartile range (IQR) and analyzed using the Kruskal–Wallis test. Categorical variables were reported as numbers (percentages) [n (%)] and compared using the χ² test. Multiple linear regression was employed to examine the cross-sectional associations between BMD T-scores and clinical/laboratory parameters. To determine the independent association of HBV DNA with BMD, three generalized additive models (GAMs) were constructed: Model 1 was unadjusted, Model 2 was adjusted for certain demographic characteristics, and Model 3 was further adjusted for all confounding factors. Smoothing curve fittings explored nonlinear associations between HBV DNA and BMD T-scores. Receiver operating characteristic (ROC) curve analysis was used to identify an optimal HBV DNA cut-off value that distinguishes patients with osteoporosis from those without, and the area under the ROC curve (AUC) was calculated to evaluate the discriminatory performance. BTMs and CRP levels among different BMD groups were compared using Kruskal–Wallis tests, whereas β-CTX and CRP differences between the high viral load group and the low viral load group were assessed via Mann–Whitney U tests. Mediation analyses using bootstrap resampling (1,000 repetitions) were conducted as an exploratory, hypothesis-generating approach to evaluate potential indirect statistical associations involving β-CTX and CRP, with 95% confidence intervals (CI) excluding zero indicating significance. All analyses were performed using SPSS 27.0.1 and Empower Stats 4.2.0. A two-tailed P-value of less than 0.05 was considered statistically significant.

## 3 Results

### 3.1 Baseline characteristics of patients with CHB

This study enrolled 362 antiviral-naive HBV patients, who were stratified into three groups based on BMD levels: normal BMD (n = 122), osteopenia (n = 136), and osteoporosis (n = 104). As summarized in **Table 1**, patients in the osteoporosis group were older than those in the normal BMD and osteopenia groups (P = 0.001). Additionally, the osteoporosis group exhibited the lowest BMI values and the highest proportion of female patients. Regarding comorbidities, hypertension was most frequent in the osteopenia group, coronary heart disease was most prevalent in the osteoporosis group, and diabetes was least common in this group. Virological analysis results demonstrated that the osteoporosis group had the highest HBV DNA levels (median: 5.70 log10 IU/mL), compared to the osteopenia group (median: 2.46 log10 IU/mL) and the normal BMD group (median: 1.83 log10 IU/mL, P < 0.001). However, among groups, no significant differences were observed in HBeAg or HBcAb levels. Liver function parameters, including albumin, aspartate aminotransferase (AST), alanine aminotransferase (ALT), alkaline phosphatase (ALP), and total bilirubin, showed no significant intergroup differences. However, the osteoporosis group had the lowest low-density lipoprotein cholesterol (LDL-C) and triglyceride levels and the highest high-density lipoprotein cholesterol (HDL-C) levels. Additionally, serum phosphorus levels were found to be inversely associated with BMD levels. 25(OH)D levels were similar across the three BMD groups, with no significant between-group difference. Regarding liver fibrosis, no significant differences in the FIB-4 index were noted among the three study groups. The prevalence of cirrhosis was low in the overall cohort (13/362) and did not differ across the three groups.

**Table 1:** Baseline characteristics by BMD classification in patients with CHB.

Variable	Total (n = 362)	Normal BMD (n = 122)	Osteopenia (n = 136)	Osteoporosis (n = 104)	*P*
Age (years)	62.83 ± 8.32	61.09 ± 8.36	62.68 ± 7.56	65.08 ± 8.78	**0.001**
Sex (n, %)					<.001
Female	177 (48.90)	36 (29.51)	69 (50.74)	72 (69.23)	
Male	185 (51.10)	86 (70.49)	67 (49.26)	32 (30.77)	
BMI (kg/m2)	24.62 ± 3.20	25.18 ± 3.58	24.45 ± 3.24	24.19 ± 2.56	**0.049**
Hypertension (n, %)	174 (48.07)	44 (36.07)	77 (56.62)	53 (50.96)	**0.003**
Diabetes (n, %)	209 (57.73)	89 (72.95)	74 (54.41)	46 (44.23)	**<.001**
Coronary heart disease (n, %)	99 (27.35)	22 (18.03)	38 (27.94)	39 (37.50)	**0.005**
Smoking (n, %)	65 (17.96)	29 (23.77)	22 (16.18)	14 (13.46)	0.104
Alcohol intake (n, %)	65 (17.96)	38 (31.15)	18 (13.24)	9 (8.65)	**<.001**
Calcium supplements (n, %)	2 (0.55)	1 (0.82)	0 (0.00)	1 (0.96)	0.530
Vitamin D supplements (n, %)	2 (0.55)	1 (0.82)	0 (0.00)	1 (0.96)	0.530
HBV DNA level (log_10_ IU/mL)	2.28 (1.81–5.28)	1.83 (1.72–2.61)	2.46 (1.88–4.80)	5.70 (2.02–6.82)	**<.001**
HBeAg	0.38 (0.28–0.55)	0.38 (0.32–0.54)	0.39 (0.31–0.54)	0.35 (0.24–0.76)	0.344
HBcAb	8.66 (7.18–9.87)	8.14 (7.16–9.46)	8.75 (6.83–9.98)	8.80 (7.67–9.69)	0.166
Albumin (g/L)	46.18 ± 9.22	46.25 ± 8.63	46.81 ± 9.68	45.29 ± 9.29	0.447
Aspartate aminotransferase (U/L)	20.90 (16.00–29.00)	20.50 (17.00–28.00)	21.00 (16.20–28.00)	19.90 (15.00–31.00)	0.591
Alanine aminotransferase (U/L)	21.80 (15.33–33.22)	22.00 (15.25–31.00)	21.75 (15.70–32.02)	20.90 (14.93–36.00)	0.865
Alkaline phosphatase (U/L)	73.62 ± 30.88	72.05 ± 33.33	73.15 ± 30.40	76.08 ± 28.57	0.606
Total bilirubin (μmol/L)	13.60 (10.40–16.99)	14.61 (10.41–18.20)	13.34 (10.45–18.23)	13.32 (10.29–15.87)	0.445
Total cholesterol (mmol/L)	4.32 (3.53–5.04)	4.39 (3.55–4.87)	4.27 (3.51–5.06)	4.31 (3.58–5.04)	0.875
LDL-C (mmol/L)	2.67 (2.23–3.13)	2.76 (2.12–3.06)	2.82 (2.33–3.28)	2.61 (2.21–3.11)	**0.041**
HDL-C (mmol/L)	1.29 (1.05–1.45)	1.26 (1.01–1.38)	1.26 (1.05–1.42)	1.31 (1.08–1.57)	**0.037**
Triglycerides (mmol/L)	1.19 (0.79–1.52)	1.23 (0.79–1.59)	1.28 (0.87–1.59)	1.06 (0.73–1.39)	**0.045**
Fasting glucose (mmol/L)	5.98 (5.05–6.87)	6.25 (5.18–7.79)	5.89 (5.07–6.67)	5.73 (4.86–6.40)	0.092
Phosphorus (mmol/L)	1.13 (1.04–1.25)	1.19 (1.05–1.29)	1.14 (1.07–1.26)	1.10 (0.99–1.15)	**<.001**
Total calcium (mmol/L)	2.28 (2.22–2.36)	2.28 (2.22–2.37)	2.29 (2.22–2.35)	2.27 (2.18–2.33)	0.180
Creatinine (μmol/L)	70.23 ± 25.15	69.90 ± 18.67	70.00 ± 28.93	70.92 ± 26.64	0.946
Platelets (×10^9^/L)	234.66 ± 49.01	234.41 ± 49.54	229.47 ± 47.73	241.75 ± 49.64	0.157
25(OH)D (ng/mL)	15.34 (10.70–20.20)	15.40 (10.94–20.30)	15.21 (10.46–20.46)	15.26 (10.78–19.80)	0.960
FIB-4	1.25 (1.09–1.44)	1.26 (1.04–1.47)	1.23 (1.09–1.44)	1.23 (1.14–1.42)	0.774
Cirrhosis (n, %)	13 (3.59)	5 (4.10)	2 (1.47)	6 (5.77)	0.179

BMI, body mass index; HBeAg, hepatitis B e antigen; HBcAb, hepatitis B core antibody; LDL-C, low-density lipoprotein cholesterol; HDL-C, high-density lipoprotein cholesterol; 25(OH)D, 25-hydroxyvitamin D; FIB-4, fibrosis-4 index.

Bold values indicate statistical significance (P < 0.05).

### 3.2 Association between HBV DNA and BMD

Significant variables identified by univariate regression in the total cohort and in male and female subgroups (**Supplementary Table 1**) were entered into multivariable regression models for further analysis. In multivariable regression (**Table 2**) HBV DNA levels were independently associated with lower BMD T-score. In the total cohort, a significant and independent negative association was observed between HBV DNA levels and BMD T-score, with each 1 log_10_ IU/mL increase in HBV DNA corresponding to a 0.22-unit reduction in BMD T-score (β = −0.22, P < 0.001). Notably, this association exhibited gender-specific differences: the strength of the association in males (β = −0.32, P < 0.001) was approximately 2-fold greater than in females (β = −0.16, P < 0.001). Furthermore, male gender was independently associated with higher BMD T-score, while age was independently associated with lower BMD T-score only in females (β = −0.02, P = 0.015). Hypertension showed a moderate negative association with BMD T-score (β = -0.29, P = 0.029), and this association lost significance upon gender stratification, while diabetes maintained a positive association with BMD T-score across both male and female participants. LDL-C was associated with higher BMD T-score only in females. Notably, HDL-C was negatively associated with BMD T-score in male participants, but no association was observed in the total cohort and in females. Additionally, serum calcium levels were positively correlated with BMD T-score in the total cohort and in females.

**Table 2:** Multivariable linear regression analysis of factors associated with BMD T-scores.

Population	Total			Male*			Female**		
Variables	β	t	*P*	β	t	*P*	β	t	*P*
Male (n, %)	0.43	3.38	**<.001**						
Age (years)	-0.01	-1.31	0.191				-0.02	-2.45	**0.015**
BMI (kg/m^2^)	0.03	1.42	0.157	0.03	1.09	0.279			
Smoking (n, %)							-0.11	-0.11	0.913
Alcohol intake (n, %)	0.10	0.59	0.554				1.82	1.61	0.110
Hypertension (n, %)	-0.29	-2.20	**0.029**						
Diabetes (n, %)	0.59	4.74	**<.001**	0.45	2.64	**0.009**	0.46	2.98	**0.003**
Coronary heart disease (n, %)	-0.19	-1.29	0.198						
HBV DNA level (log_10_ IU/mL)	-0.22	-8.00	**<.001**	-0.32	-7.15	**<.001**	-0.16	-4.84	**<.001**
HBeAg				-0.00	-0.80	0.422			
LDL-C (mmol/L)							0.21	2.26	**0.025**
HDL-C (mmol/L)	-0.30	-1.73	0.085	-0.65	-2.70	**0.008**			
Triglycerides (mmol/L)	0.05	0.78	0.438						
Phosphorus (mmol/L)	0.47	1.88	0.061	0.47	1.44	0.150			
Total calcium (mmol/L)	1.10	2.73	**0.007**	0.52	0.94	0.351	1.51	2.67	**0.008**
Platelets (×109/L)							-0.00	-1.55	0.124

Male*: Male population (age ≥50 years); Female**: Postmenopausal female population.

BMI, body mass index; HBeAg, hepatitis B e antigen; LDL-C, low-density lipoprotein cholesterol; HDL-C, high-density lipoprotein cholesterol.

Bold values indicate statistical significance (P < 0.05).

### 3.3 Negative correlation between HBV DNA and BMD

**Figure 2:**
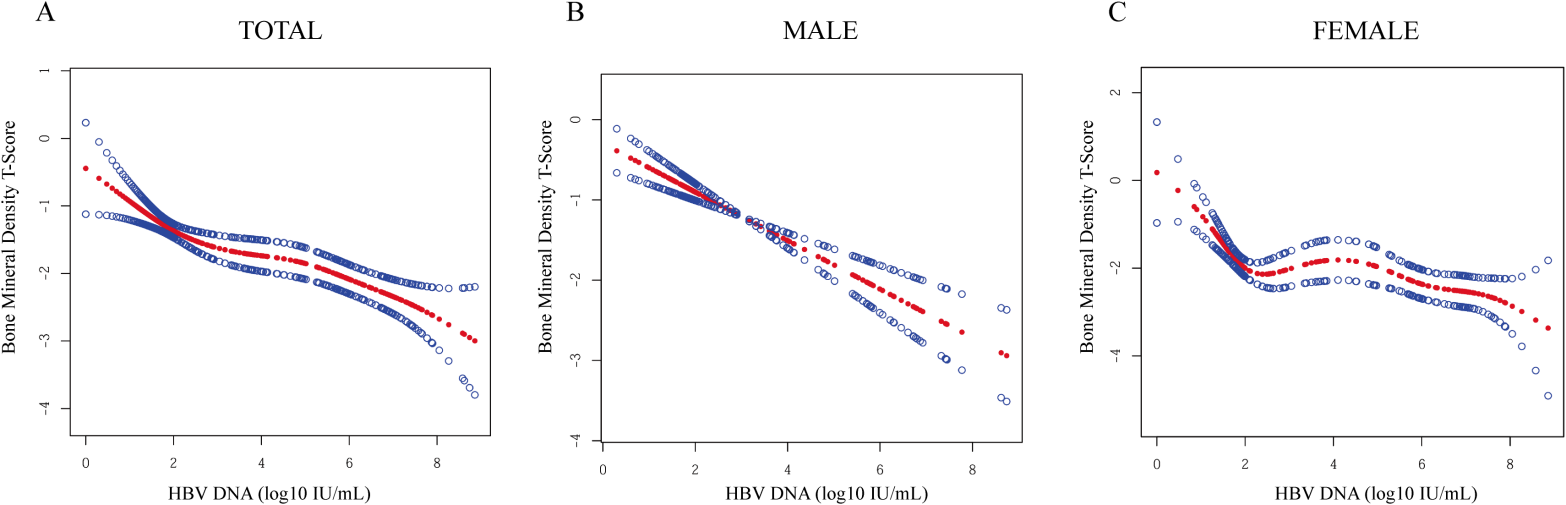
Relationships between HBV DNA levels and BMD T-scores. **(A)** Total: Middle-aged and elderly antiviral-naive patients with CHB. **(B)** Male: Male population (age ≥50 years). **(C)** Female: Postmenopausal female population. Solid red curves depict smoothed fit from generalized additive mixed models, with blue lines indicating 95% confidence intervals.

The association between HBV DNA levels and BMD T-scores was systematically evaluated using GAMs. In unadjusted Model 1, HBV DNA levels showed a significant negative correlation with BMD (β = -0.274, P <0.001). Further stratified analysis revealed that this negative association remained significant in the male (β = -0.358, P <0.001) and female subgroups (β = -0.175, P <0.001). In Model 2, after adjusting for demographic characteristics (age, BMI, and gender in the total population), the negative correlation was still significant in all three study populations (all P < 0.05). In the fully adjusted Model 3, which accounted for all the potential confounders, the results further confirmed an independent negative association of HBV DNA with BMD T-scores. Specifically, for each 1 log_10_ IU/mL increase in HBV DNA levels, there was a -0.207-unit decrease in BMD T-score within the total study population, with reductions of -0.285 in males and -0.172 in females (**Table 3**). Higher HBV DNA levels were associated with progressively worse BMD in the three groups. These results indicate that the detrimental association between HBV DNA levels and BMD is not restricted to a specific diagnostic threshold but is evident across the continuum from normal bone density to osteopenia and osteoporosis. Finally, smoothing curve fitting (**Figure 2**) revealed a nonlinear relationship between HBV DNA levels and BMD T-scores.

**Table 3:** Association between HBV DNA and BMD T-scores.

		Model 1		Model 2		Model 3	
Population	BMD	β (95% CI)	*P*	β (95% CI)	*P*	β (95% CI)	*P*
	Overall	-0.274 (-0.331, -0.217)	**<0.001**	-0.237 (-0.293, -0.181)	**<0.001**	-0.207 (-0.264, -0.150)	**<0.001**
	Normal BMD	Reference		Reference		Reference	
Total	Osteopenia	1.035 (0.581, 1.488)	**<0.001**	0.978 (0.510, 1.445)	**<0.001**	0.908 (0.418, 1.397)	**<0.001**
	Osteoporosis	2.555 (2.070, 3.041)	**<0.001**	2.450 (1.924, 2.976)	**<0.001**	2.328 (1.756, 2.899)	**<0.001**
	P for trend	**<0.001**		**<0.001**		**<0.001**	
	Overall	-0.358 (-0.446, -0.269)	**<0.001**	-0.354 (-0.443, -0.266)	**<0.001**	-0.285 (-0.388, -0.183)	**<0.001**
	Normal BMD	Reference		Reference		Reference	
Male*	Osteopenia	1.066 (0.564, 1.568)	**<0.001**	1.112 (0.598, 1.626)	**<0.001**	0.965 (0.433, 1.497)	**<0.001**
	Osteoporosis	2.835 (2.197, 3.473)	**<0.001**	2.850 (2.198, 3.502)	**<0.001**	2.264 (1.520, 3.007)	**<0.001**
	P for trend	**<0.001**		**<0.001**		**<0.001**	
	Overall	-0.175 (-0.245, -0.106)	**<0.001**	-0.157 (-0.226, -0.088)	**<0.001**	-0.172 (-0.243, -0.102)	**<0.001**
	Normal BMD	Reference		Reference		Reference	
Female**	Osteopenia	0.878 (0.024, 1.732)	**0.045**	0.900 (0.023, 1.777)	**0.044**	0.886 (-0.090, 1.861)	0.075
	Osteoporosis	2.258 (1.410, 3.106)	**<0.001**	2.225 (1.333, 3.117)	**<0.001**	2.496 (1.503, 3.490)	**<0.001**
	P for trend	**<0.001**		**<0.001**		**<0.001**	

Male*: Male population (age ≥50 years); Female**: Postmenopausal female population.

Model 1: Unadjusted.

Model 2: Adjusted for age and BMI (additionally for gender in the total population).

Model 3: Adjusted for age; BMI; hypertension; diabetes; coronary heart disease; smoking; alcohol intake; hepatitis B e antigen; hepatitis B core antibody; albumin; aspartate aminotransferase; alanine aminotransferase; alkaline phosphatase; total bilirubin; total cholesterol; low-density lipoprotein cholesterol; high-density lipoprotein cholesterol; triglycerides; fasting glucose; phosphorus; total calcium; creatinine; platelet; 25-hydroxyvitamin D; fibrosis-4 index; cirrhosis.

Bold values indicate statistical significance (P < 0.05).

### 3.4 Discriminatory ability of HBV DNA levels for osteoporosis

**Figure 3:**
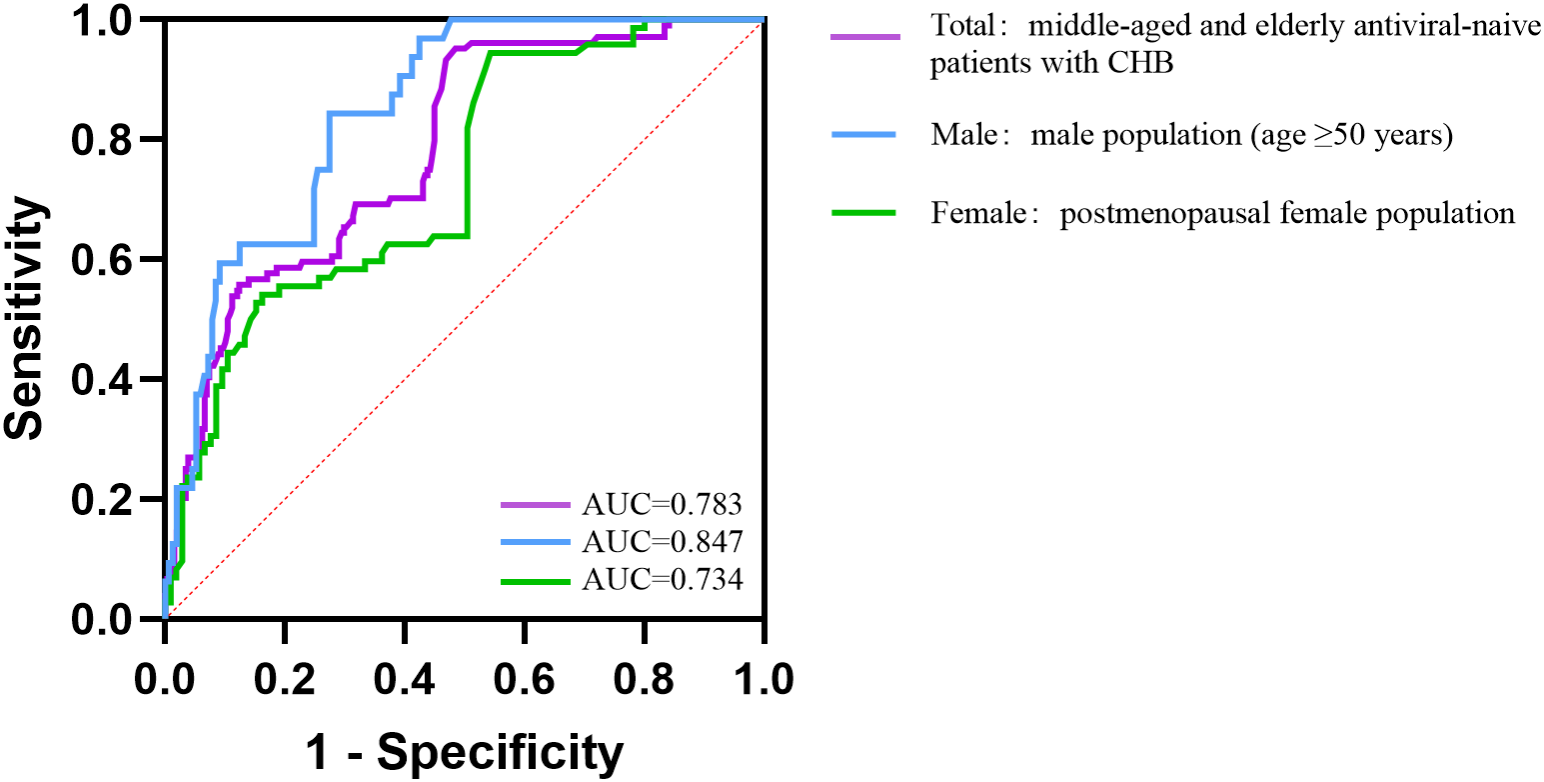
ROC curves of HBV DNA for distinguishing osteoporosis. The area under curve (AUC) was 0.783 in the total population, 0.847 in the male population and 0.734 in the female population.

ROC curve analysis was used to assess the discriminatory ability of HBV DNA and to identify cut-off values for differentiating patients with osteoporosis from those without across study groups (**Figure 3**). In the total population, the AUC for HBV DNA levels was 0.783 (95% CI: 0.733-0.833; **Table 4**), with an optimal cut-off value of 1.952 log10 IU/mL and a Youden index of 0.475. HBV DNA showed greater discriminatory performance in males (AUC = 0.847, 95% CI: 0.785-0.909) than in females (AUC = 0.734, 95% CI: 0.660-0.808). In males, the optimal cut-off value of HBV DNA was 2.857 log10 IU/mL, and the Youden index reached 0.569 (**Table 4**). These results indicate that HBV DNA load has a significant ability to distinguish individuals with osteoporosis from those without, especially in middle-aged and older men.

**Table 4:** Performance of HBV DNA in distinguishing osteoporosis.

Population	Youden index	AUC	95% CI	Cut−Off
Total	0.475	0.783	0.733 – 0.833	1.952
Male^*^	0.569	0.847	0.785 – 0.909	2.857
Female^**^	0.401	0.734	0.660 – 0.808	1.947

Male*: Male population (age ≥50 years); Female**: Postmenopausal female population.

Cut-off values are expressed in log10 IU/mL of HBV DNA.

### 3.5 Potential mediating roles of β-CTX and CRP

We found statistical significance for the BTM β-CTX and the inflammatory marker CRP when comparing patient data across different BMD groups (**Table 5**). Specifically, the osteoporosis group had higher β-CTX levels (0.61; 0.36–1.07 ng/mL) compared to both the osteopenia group (0.47; 0.36–0.69 ng/mL) and normal BMD group (0.35; 0.25–0.48 ng/mL). Similarly, the highest CRP level was observed in the osteoporosis group (3.00; 2.00–4.92 mg/L). No statistical difference was observed for P1NP, N-MID, OC, 25(OH)D, or PTH among groups.

**Table 5:** Clinical and biochemical variables in patients with normal BMD, osteopenia, and osteoporosis.

Variables	Normal BMD (n = 122)	Osteopenia (n = 136)	Osteoporosis (n = 104)	*P*
β-CTX (ng/mL)	0.35 (0.25–0.48)	0.47 (0.36–0.69)	0.61 (0.36–1.07)	**<.001**
P1NP (ng/mL)	38.20 (33.23–54.98)	43.35 (35.27–53.10)	43.30 (32.00–55.65)	0.343
N-MID (ng/mL)	15.17 (12.30–18.10)	15.10 (12.07–17.35)	15.64 (10.69–19.02)	0.955
OC (ng/mL)	16.52 (6.83–21.77)	16.40 (10.34–22.19)	16.35 (8.62–20.73)	0.688
25(OH)D (ng/mL)	15.40 (10.94–20.30)	15.21 (10.46–20.46)	15.26 (10.78–19.80)	0.960
PTH (pg/mL)	29.25 (22.41–39.58)	31.00 (24.62–39.50)	31.19 (23.75–43.67)	0.484
CRP (mg/L)	1.17 (0.70–2.10)	2.16 (1.07–3.96)	3.00 (2.00–4.92)	**<.001**

β-CTX, β-C-terminal telopeptide of type I collagen; P1NP, procollagen type I N-propeptide; N-MID, N-terminal mid-fragment osteocalcin; OC, osteocalcin; 25(OH)D, 25-hydroxyvitamin D; PTH, parathyroid hormone; CRP, C-reactive protein.

Bold values indicate statistical significance (P < 0.05).

**Figure 4:**
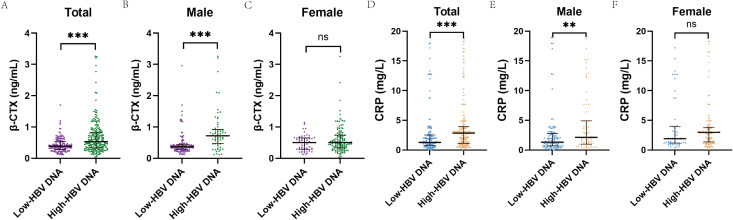
Comparison of β-CTX and CRP levels between high and low viral load groups. **(A)** The β-CTX level was significantly higher in the group with high HBV DNA load in the total population. **(B)** The β-CTX level was significantly higher in the group with high HBV DNA load in the male population. **(C)** The difference in β-CTX levels between the high and low HBV DNA load groups was insignificant in the female population. **(D)** The CRP level was significantly higher in the group with high HBV DNA load in the total population. **(E)** The CRP level was significantly higher in the group with high HBV DNA load in the male population. **(F)** The difference in CRP levels between the high and low HBV DNA load groups was insignificant in the female population. Total: Middle-aged and elderly antiviral-naive patients with CHB; Male: Male population (age ≥50 years); Female: Postmenopausal female population. ***P < 0.001, **P < 0.01, n.s., not significant (P > 0.05).

Next, we established HBV DNA cut-off values (total study population: 1.952 log_10_ IU/mL; males: 2.857 log_10_ IU/mL; females: 1.947 log_10_ IU/mL) through ROC curve analysis and using these thresholds, we categorized participants into high viral load and low viral load groups to compare β-CTX and CRP levels. β-CTX and CRP levels were significantly elevated in the high viral load groups of the total and male cohorts (**Figure 4**), although the difference was not significant in females.

**Figure 5:**
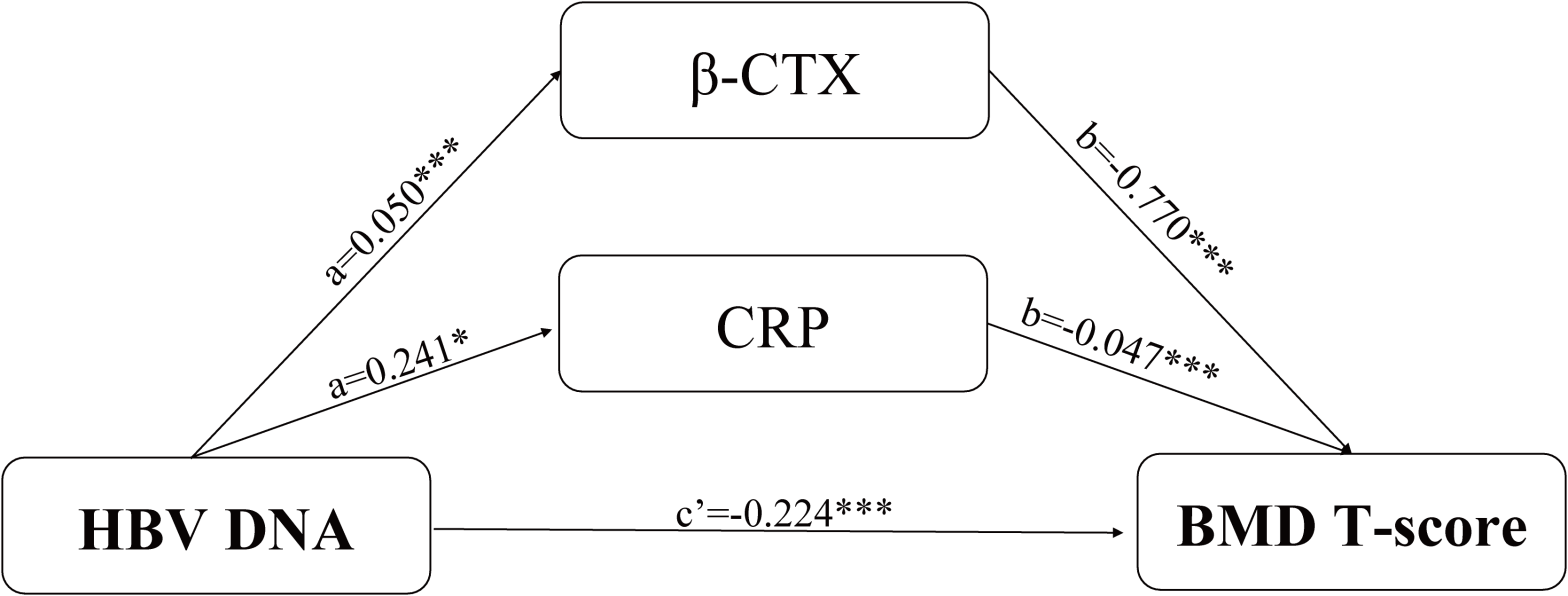
Mediation model linking HBV DNA to BMD T-score in the total population. The analysis suggested exploratory indirect associations involving β-CTX and CRP in the association between HBV DNA and BMD T-score. A direct association between HBV DNA and BMD T-score was also observed. ***P < 0.001, *P < 0.05.

We conducted an exploratory mediation analysis to examine putative indirect associations underlying the association between HBV DNA and BMD. According to the results above, we selected β-CTX and CRP as candidate mediators. In the total study population, the path coefficients supported exploratory indirect associations via β-CTX and CRP (**Table 6**): higher HBV DNA was associated with increased β-CTX levels (Path a = 0.050, P < 0.001), which in turn were associated with lower BMD T-scores (Path b = -0.770, P < 0.001). Similarly, higher HBV DNA was associated with elevated CRP levels (Path a = 0.241, P = 0.021), and elevated CRP was subsequently associated with lower BMD T-scores (Path b = -0.047, P < 0.001). In male participants, β-CTX demonstrated an even more pronounced pattern of mediation, whereas the mediation role of CRP was statistically excluded (P = 0.169). In female participants, neither β-CTX nor CRP showed a statistically significant mediating function. Notably, the direct association (c’) of HBV DNA with BMD T-scores remained pronounced in all models. The conceptual diagram shown in **Figure 5** illustrates these exploratory indirect associations potentially linking HBV DNA to BMD T-scores through β-CTX and CRP, as well as the direct association of HBV DNA with BMD T-scores.

**Table 6:** Mediation analysis of HBV DNA and BMD.

Group	Mediator	Path a (X→M)	*P*	Path b (M→Y)	*P*	Direct effect (c’)	*P*
Total	β-CTX (ng/mL)	0.050	**<0.001**	-0.770	**<0.001**	-0.224	**<0.001**
	CRP (mg/L)	0.241	**0.021**	-0.047	**<0.001**	-0.224	**<0.001**
Male*	β-CTX (ng/mL)	0.093	**<0.001**	-0.841	**<0.001**	-0.267	**<0.001**
	CRP (mg/L)	0.507	**0.003**	-0.027	0.169	-0.267	**<0.001**
Female**	β-CTX (ng/mL)	0.024	0.060	-0.795	**<0.001**	-0.154	**<0.001**
	CRP (mg/L)	0.032	0.809	-0.056	**0.003**	-0.154	**<0.001**

Male*: Male population (age ≥50 years); Female**: Postmenopausal female population.

X: HBV DNA (log_10_ IU/mL); Y: BMD T-score.

Mediators (M): β-CTX, β-C-terminal telopeptide of type I collagen; CRP, C-reactive protein.

Path a: Effect of X on mediator; Path b: Effect of mediator on Y (adjusted for X); c’: Direct effect of X on Y (controlling for mediators).

Bold values indicate statistical significance (P < 0.05).

## 4 Discussion

This cross-sectional study involving 362 antiviral-naive middle-aged and elderly patients with CHB systematically demonstrates, for the first time, that higher HBV DNA levels are correlated with lower BMD across the continuum from normal bone density to osteoporosis. Notably, HBV DNA exhibited a strong between-group discriminatory ability to distinguish patients with osteoporosis, especially in males. Exploratory mediation analysis raises the hypothesis that β-CTX and CRP may act as mediators in the association between HBV DNA and BMD. This study provides evidence that higher HBV DNA levels are independently associated with osteoporosis in untreated patients with CHB, and identifies specific viral load thresholds that warrant further investigation. Our findings highlight the potential clinical relevance of HBV DNA levels in the assessment of bone health in patients with CHB and suggest that, as a routine clinical parameter, HBV DNA may aid in identifying individuals at risk for bone loss. Subject to future longitudinal confirmation, HBV DNA thresholds could inform strategies for fracture risk reduction and alleviation of disease burden in patients with CHB.

Previous studies have consistently shown a significantly higher prevalence of osteoporosis and fractures among patients with CHB (23–25) compared to the general population. However, the direct association between HBV DNA and BMD remains inconclusive. Dessordi et al. conducted a cross-sectional DXA-based study in 81 adults with CHB infection who were stratified by antiviral exposure. In multivariable models, higher HBV load was independently associated with reduced LS BMD (β = −0.081, P = 0.01) and TH BMD (β = −0.047, P = 0.04) among untreated carriers, whereas these associations were not evident in treated patient groups (26). By investigating the association between HBV DNA levels and BMD in treatment-naive patients with CHB, our study avoids the potential confounding of antiviral medications, providing evidence that high HBV DNA levels are associated with lower BMD T-scores. This association is further supported by previous interventional studies. Ogawa et al. demonstrated that achieving and maintaining profound viral suppression (HBV DNA <20 IU/mL) through antiviral therapy was associated with a significant improvement in spinal BMD (27). In addition, a recent large-scale prospective cohort study from the Hong Kong Osteoporosis Study demonstrated that low BMD could be a novel risk factor and early predictor for cirrhosis (28). Collectively, these findings highlight an interplay between liver disease and bone health, although the directionality and underlying mechanisms require further longitudinal investigation.

In our fully adjusted model, each 1 log_10_ IU/mL increase in HBV DNA corresponded to an approximately 0.207-unit decrease in BMD T-score, representing a modest shift in the T-score metric. From a clinical perspective, prior prospective evidence indicates that a 1 SD lower BMD is associated with an increased fracture risk, with relative risks of approximately 1.5 for osteoporotic fractures overall, and higher estimates for site-specific outcomes such as vertebral fractures (≈2.3) and hip fractures (≈2.6) (29). Nevertheless, because the present study did not collect incident fracture outcomes or apply validated risk prediction models, the extent to which the observed BMD differences translate into fracture risk in patients with CHB remains uncertain. Because our findings derive from cross-sectional associations, they should not be regarded as direct evidence for fracture prevention strategies. In addition, both the magnitude and shape of the HBV–BMD association appeared to differ by sex, a pattern that has been widely reported but remains inconsistent across studies. Chen et al. found an inverse association between HBV infection and BMD in males, but no significant association in females (30), whereas analysis of a large German real-world cohort reported increased osteoporosis risk mainly in women (12). In our cohort, HBV DNA was inversely associated with BMD in both sexes, yet the effect was substantially stronger in males (β = −0.32) than in postmenopausal females (β = −0.16). This disparity may plausibly reflect sex-hormone modulation of viral replication and bone remodeling: androgen receptor signaling can enhance HBV transcription (31), while estrogen/ERα may repress HBV gene expression, potentially lowering viral burden in women (32). Additionally, estrogen deficiency promotes osteoclast activity via RANKL-related pathways, which may attenuate or obscure additional cross-sectional associations of viral load in postmenopausal women (33). Accordingly, our GAM analyses showed a smooth, near-linear dose–response pattern between HBV DNA and BMD T-score in males but a fluctuating trend in females. Notably, the apparent rebound around HBV DNA ≈ 4 log10 IU/mL was likely due to the sparse data of this range and local instability of smoothing estimates, rather than a true biological reversal in females.

In this cross-sectional analysis, findings from ROC analysis should be interpreted as exploratory, reflecting between-group discriminatory ability (osteoporosis vs non-osteoporosis) rather than diagnostic or prospective predictive performance; accordingly, the sex-specific cut-offs reported here should be viewed as hypotheses requiring validation in independent, prospective cohorts. Notably, comparable ROC-based discrimination using HBV DNA in antiviral-naive CHB populations has been rarely described; prior work has more often focused on cohorts under antiviral treatment or on patients with hepatic osteodystrophy, a metabolic bone disorder linked to chronic liver disease, most commonly observed in patients with cirrhosis. For example, in men with HBV-related chronic liver disease, high viral load (>10,000 IU/mL) was associated with low FN bone mass (34); however, concomitant tenofovir exposure (a known risk factor for reduced BMD) and study design limit direct comparability with our untreated cohort. Other discrimination approaches, such as the FRAX tool, have been evaluated in CHB. In tenofovir-treated cohorts, FRAX showed high AUC for identifying individuals meeting treatment thresholds, underscoring that multifactorial models may outperform single biomarkers in fracture-risk assessment (35, 36).

Beyond HBV DNA, our biomarker comparisons provide additional exploratory signals: β-CTX and CRP showed clear gradients across BMD status, peaking in the osteoporosis group, whereas bone formation or mixed remodeling markers (P1NP, osteocalcin/N-MID) and calcium–phosphate regulatory markers (25(OH)D, PTH) did not differ significantly. Furthermore, when participants were stratified by ROC-derived HBV DNA cut-offs, β-CTX and CRP were higher in the high viral load group in the total and male cohorts but not in females. Our exploratory mediation analysis suggested potential indirect statistical associations between HBV DNA and BMD via β-CTX and CRP in the total study population (with evidence mainly for β-CTX in males and no significant evidence of mediation in females), whereas the direct HBV DNA–BMD association was consistently significant across models. However, the results of this mediation analysis remain hypothetical and require further biological and clinical evidence (e.g., cytokine panels, sex hormone measurements, detailed metabolic profiling) to be confirmed. Given that β-CTX is a well-established marker of type I collagen degradation and is widely used as a reference indicator of bone resorption (37), a possible biological interpretation consistent with existing literature is that higher HBV replication may coincide with heightened immune activation and inflammatory signaling (38), which could promote osteoclastogenesis via RANKL/NF-κB pathways and thereby increase bone resorption with elevated β-CTX (39). CRP is a liver-derived acute-phase reactant. Experimental evidence suggests HBV can upregulate CRP expression (40), and epidemiologic data further link higher CRP to adverse skeletal outcomes, including fracture risk (41). Although our mediation results remain preliminary and require mechanistic confirmation, the loss of significance in sex-stratified models may reflect limited subgroup power and residual confounding (e.g., heterogeneity in postmenopausal status, unmeasured sex hormones, body fat distribution) rather than a true absence of indirect associations.

Although our study provides novel evidence of a robust association between HBV DNA levels and BMD in patients with CHB across the bone mineral density spectrum, several limitations must be acknowledged. First, the single-center design may introduce potential biases related to region, ethnicity, or clinical practice; therefore, future multicenter studies would be beneficial in validating our findings. Second, its cross-sectional nature precludes causal inference, and the observed associations may be influenced by unmeasured confounding from factors such as liver stiffness and key metabolic indicators, including sex hormone levels. Although we incorporated 25-hydroxyvitamin D in the fully adjusted models, vitamin D status may still be incompletely captured in this cross-sectional setting, and residual confounding by vitamin D–related factors cannot be excluded. Therefore, these findings require validation and their causality should be assessed through future prospective cohort or intervention studies that incorporate such measurements. Furthermore, though we identified β-CTX and CRP as candidate mediators in exploratory analyses of the association between HBV DNA and BMD, given the cross-sectional design, these mediation signals should be interpreted as exploratory and hypothesis-generating. Future molecular studies are warranted to validate these hypothesized associations and clarify their biological relevance.

## 5 Conclusion

In conclusion, this study reports an independent inverse association between HBV DNA levels and BMD in antiviral-naive middle-aged and elderly patients with CHB. In addition, β-CTX and CRP are identified as potential mediators in exploratory analyses in the association between HBV DNA and BMD. These findings highlight the necessity for clinicians to monitor bone metabolism in patients with CHB with elevated HBV DNA levels and consider appropriate screening and risk assessment.

## Data Availability

The raw data supporting the conclusions of this article will be made available by the authors, without undue reservation.
