# Supplementary material for: Association between HBV DNA levels and bone mineral density in antiviral-naive chronic hepatitis B patients
Source: Front Endocrinol (Lausanne). 2026 Feb 18;17:1714943. doi: 10.3389/fendo.2026.1714943 (PMC12956655; doi:10.3389/fendo.2026.1714943)
Supplement: Supplementary file 1 [file Table1.docx]

Supplementary Material

**Supplementary Table 1.** Univariate linear regression analyses.

|  | Total | | |  | Male^*^ | | |  | Female^**^ | | |
| --- | --- | --- | --- | --- | --- | --- | --- | --- | --- | --- | --- |
| Variables | β | t | *P* |  | β | t | *P* |  | β | t | *P* |
| Male (n, %) | 0.790 | 6.090 | **<.001** |  |  |  |  |  |  |  |  |
| Age (years) | -0.024 | -3.022 | **0.003** |  | -0.004 | -0.318 | 0.751 |  | -0.033 | -3.522 | **<.001** |
| BMI (kg/m2) | 0.057 | 2.686 | **0.008** |  | 0.068 | 2.383 | **0.018** |  | 0.042 | 1.470 | 0.143 |
| Hypertension (n, %) | -0.322 | -2.384 | **0.018** |  | -0.113 | -0.571 | 0.569 |  | -0.283 | -1.646 | 0.102 |
| Diabetes (n, %) | 0.602 | 4.486 | **<.001** |  | 0.672 | 3.457 | **<.001** |  | 0.381 | 2.244 | **0.026** |
| Coronary heart disease (n, %) | -0.381 | -2.513 | **0.012** |  | -0.244 | -1.038 | 0.301 |  | -0.289 | -1.594 | 0.113 |
| Smoking (n, %) | 0.235 | 1.329 | 0.185 |  | -0.348 | -1.699 | 0.091 |  | 1.235 | 2.162 | **0.032** |
| Alcohol intake (n, %) | 0.694 | 3.998 | **<.001** |  | 0.225 | 1.096 | 0.274 |  | 1.703 | 2.604 | **0.010** |
| Calcium supplements (n, %) | -0.097 | -0.106 | 0.916 |  |  |  |  |  |  |  |  |
| Vitamin D (n, %) | -0.097 | -0.106 | 0.916 |  |  |  |  |  |  |  |  |
| HBV DNA level (log_10_ IU/mL) | -0.274 | -9.467 | **<.001** |  | -0.358 | -7.957 | **<.001** |  | -0.175 | -4.967 | **<.001** |
| HBeAg | -0.001 | -1.662 | 0.097 |  | -0.001 | -2.133 | **0.034** |  | -0.000 | -0.033 | 0.974 |
| HBcAb | -0.033 | -1.377 | 0.170 |  | -0.046 | -1.445 | 0.150 |  | -0.000 | -0.005 | 0.996 |
| Total cholesterol (mmol/L) | 0.028 | 0.663 | 0.508 |  | 0.023 | 0.346 | 0.730 |  | 0.010 | 0.218 | 0.827 |
| LDL-C (mmol/L) | 0.069 | 0.828 | 0.408 |  | 0.009 | 0.078 | 0.938 |  | 0.244 | 2.319 | **0.022** |
| HDL-C (mmol/L) | -0.623 | -3.291 | **0.001** |  | -0.814 | -3.016 | **0.003** |  | -0.024 | -0.098 | 0.922 |
| Triglycerides (mmol/L) | 0.159 | 2.124 | **0.034** |  | 0.103 | 1.100 | 0.273 |  | 0.203 | 1.783 | 0.076 |
| Total bilirubin (μmol/L) | 0.000 | 0.088 | 0.930 |  | 0.001 | 0.157 | 0.876 |  | -0.002 | -0.404 | 0.687 |
| Albumin (g/L) | 0.009 | 1.208 | 0.228 |  | 0.006 | 0.563 | 0.574 |  | 0.014 | 1.496 | 0.136 |
| Aspartate aminotransferase (U/L) | -0.000 | -0.052 | 0.958 |  | 0.004 | 1.095 | 0.275 |  | -0.003 | -1.423 | 0.157 |
| Alanine aminotransferase (U/L) | 0.001 | 0.523 | 0.601 |  | 0.001 | 0.573 | 0.567 |  | -0.002 | -1.194 | 0.234 |
| Alkaline phosphatase (U/L) | -0.003 | -1.429 | 0.154 |  | -0.004 | -1.255 | 0.211 |  | -0.004 | -1.149 | 0.252 |
| Creatinine (μmol/L) | -0.000 | -0.166 | 0.868 |  | -0.005 | -1.157 | 0.249 |  | -0.000 | -0.059 | 0.953 |
| Fasting glucose (mmol/L) | 0.029 | 0.971 | 0.332 |  | -0.006 | -0.142 | 0.887 |  | 0.055 | 1.367 | 0.173 |
| Phosphorus (mmol/L) | 0.655 | 2.231 | **0.026** |  | 0.881 | 2.321 | **0.021** |  | 0.467 | 1.118 | 0.265 |
| Total calcium (mmol/L) | 1.407 | 2.952 | **0.003** |  | 1.420 | 2.225 | **0.027** |  | 1.418 | 2.195 | **0.029** |
| Platelets (×10^9^/L) | -0.002 | -1.262 | 0.208 |  | 0.001 | 0.552 | 0.582 |  | -0.005 | -2.680 | **0.008** |
| 25(OH)D (ng/mL) | -0.007 | -0.772 | 0.440 |  | 0.009 | 0.584 | 0.560 |  | -0.008 | -0.795 | 0.427 |
| FIB-4 | 0.020 | 0.176 | 0.861 |  | 0.180 | 1.193 | 0.234 |  | -0.218 | -1.350 | 0.179 |
| Cirrhosis (n, %) | -0.242 | -0.662 | 0.508 |  | -0.003 | -0.006 | 0.995 |  | -0.841 | -1.632 | 0.105 |

Male*: Male population (age ≥50 years); Female**: Postmenopausal female population.

Bold values indicate statistical significance (P < 0.05).

BMI, body mass index; HBeAg, hepatitis B e antigen; HBcAb, hepatitis B core antibody; LDL-C, low-density lipoprotein cholesterol; HDL-C, high-density lipoprotein cholesterol; 25(OH)D, 25-hydroxyvitamin D; FIB-4, fibrosis-4 index.
